# Market concentration and the healthiness of packaged food and non-alcoholic beverage sales across the European single market

**DOI:** 10.1017/S1368980022001926

**Published:** 2022-11

**Authors:** Iris Van Dam, Olivier Allais, Stefanie Vandevijvere

**Affiliations:** 1 Université Paris-Saclay, INRAE, UR ALISS, Ivry-Sur-Seine, France; 2 Sciensano, Service of Lifestyle and Chronic Diseases, Ernest Blerotstraat 1, 1070 Anderlecht, Brussels, Belgium

**Keywords:** Europe, Food industry, Food environments, Food supply, Market structure, Market power, NOVA

## Abstract

**Objective:**

To assess the relationship between market concentration and diversity, as indicators of market structure, and the healthiness of food and beverage sales across Europe.

**Design:**

Market share (MS) data per country were used to calculate market concentration, assessed by the four-firm concentration ratio and market diversity, and by the number of companies with ≥1 % MS and the number of companies uniquely present in one European country. The healthiness of food sales was assessed by applying the NOVA classification (level of processing). Simple and multiple linear regressions were performed to assess the relationship between market concentration, diversity and the healthiness of food and beverage sales.

**Setting:**

The European single market.

**Participants:**

The twenty-seven European single market member states for which Euromonitor sales data were available at the most fine-grained Euromonitor packaged food and non-alcoholic beverage product subcategory level.

**Results:**

Increased market concentration with a country and a product category fixed effect significantly predicted increased sales of ultra-processed packaged food products. There was insufficient data variability in the level of processing of non-alcoholic beverage product categories to formulate conclusions for non-alcoholic beverages. Increased market diversity in turn significantly predicted reduced country-level sales of ultra-processed products.

**Conclusions:**

The results indicated a relationship between market structure and the healthiness of packed food products sold on the European market. However, more research with detailed nutritional data is warranted to document and quantify this interaction.

Food environments are defined as ‘the collective physical, economic, policy and sociocultural surroundings, opportunities and conditions that influence people’s food and beverage choices and nutritional status’^(1)^. Currently, these environments are characterised by easily available unhealthy food products^(2–4)^ with ultra-processed foods contributing to 10 % up to 51 % of the purchased dietary energy across Europe^(5)^. Ultra-processed foods are products such as soft drinks and confectionery that contain substances that are not commonly found at home^(6)^. A growing body of literature shows an association between overweight and the consumption of such ultra-processed foods^(4,5,7,8)^. Nonetheless, ultra-processed foods are extensively promoted, with markets expanding and several political strategies being used to protect ultra-processed food markets^(9,10)^.

Market structure describes the degree at which competition takes place between different companies for specific goods and services within (product) markets^(11,12)^. A key metric to assess the market structure and power of companies is market concentration^(13)^. When concentration increases, this translates into an increasing part of the market being held by a decreasing number of companies^(4,14)^. Other market structure indicators, measuring the market diversity, are the number of companies with ≥1 % market share (MS) and the number of unique companies having presence in only one European country^(14)^.

Across countries in Europe, packaged food and non-alcoholic beverage product markets have shown to be moderately to highly concentrated with a low number of unique companies and companies with ≥1 % MS^(14)^. While the food industry publicly positions itself as part of the solution to create healthier food environments^(15,16)^, they at the same time shape markets in ways that fit their private interests^(11)^. High levels of market concentration and reduced diversity may provide dominant companies with the opportunity to shape markets in ways that benefit them financially and economically (e.g. through the increased sales of ultra-processed foods), something that does not benefit population health^(3,4,11,12,17–19)^. Examples of how the food industry may influence food environments include the framing of policy debates, intensive marketing, nutritional positioning (*i.e. focus on single nutrients instead of whole foods, an approach that could promote the sales of heavily processed foods*), focus on individual responsibility and unenforceable self-regulatory codes^(4,15,16)^. Nonetheless, research assessing the influence of market structure on food environments remains limited.

This study sets out to assess whether market structure, assessed by levels of market concentration and diversity within the packaged food and non-alcoholic beverage industry across European countries, is associated with the healthiness of products sold, measured by the proportion of sales of ultra-processed food products according to the NOVA classification.

## Methods

The Euromonitor International Passport database was used to obtain MS data per European single market member state, per packaged food and drink product category and per year^(20)^. Data were obtained at the most fine-grained Euromonitor product categorisation level over the period 2009–2018. For Cyprus, Iceland, Liechtenstein, Luxembourg and Malta, no Euromonitor data were available. A total of twenty-seven European countries were included in the analysis.

### Market concentration

Levels of market concentration and its changes over time (2009–2018) were assessed by calculating the four firm concentration ratio (CR4) per country for fourteen packaged food product markets and eight non-alcoholic beverage product markets (Table 1; Annex 1). The CR4 is calculated by combining the MS of the top four firms per country active within a product market. The higher the CR4, the more concentrated the product market. CR4 values below 40 are considered to represent a competitive market. Values above 40 are considered to represent markets with limited competition and above 60 limited competition with potential dominant firms^(21)^.

**Table 1:**
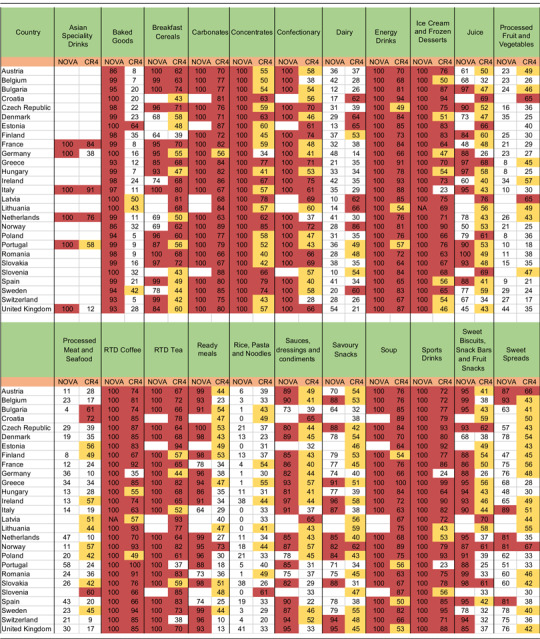
The proportion of sales from ultra-processed products (NOVA) and levels of market concentration according to the four firm concentration ratio (CR4) per country and product category. Euromonitor data 2018

Red indicates CR4 values >60 % and proportion of sales >80 %.

Yellow indicates CR4 values >40 %.

The number of companies with ≥1 % MS and the number of unique companies per country were assessed to estimate levels of diversity within packaged food and non-alcoholic beverage product markets. Unique companies were defined as companies having presence in only one European single market member state. Similar to previous research, the higher the number of companies with ≥1 % MS and unique companies, the more diverse the industry was assumed to be^(14)^.

### Products sold

To assess the proportion of sales coming from ultra-processed products, the NOVA classification^(6)^ was applied to the most fine-grained Euromonitor product subcategory sales data within abovementioned packaged food and non-alcoholic beverage product categories. An overview of how the Euromonitor product subcategories were classified according to the NOVA classification can be found in Annex 1. For five countries (Croatia, Estonia, Latvia, Lithuania and Slovenia), data were only available for the most fine-grained product subcategories within eight (out of the twenty-two) Euromonitor product categories (‘Baked Goods’, ‘Concentrates’, ‘Dairy’, ‘Energy Drinks’; ‘Ice Cream and Frozen Desserts’, ‘RTD Coffee’, ‘Rice, Pasta and Noodles’ and ‘Sports Drinks’).

The NOVA classification makes a distinction between products based on the level of processing, namely non-ultra-processed (unprocessed/minimally processed foods, processed culinary ingredients and processed foods) and ultra-processed products^(6)^. Per Euromonitor product category, the proportion of sales coming from ultra-processed subcategories was calculated by expressing the ultra-processed sales per country and product category on the total sales within the same country and product category. Finally, also the change over the past 10 years (2009–2018) of the proportion of sales coming from ultra-processed products was assessed.

### The relationship between market concentration, diversity and healthiness of packaged food and drink products sold across European countries

Analyses were conducted separately for packaged food and non-alcoholic beverage product categories. A multiple linear regression was calculated across selected countries and product categories to assess whether and to what extent market concentration measured by the CR4 influences the proportion of sales of ultra-processed products. The product categories containing 100 % ultra-processed products were removed from the analysis. Among packaged food products these were ‘Confectionary’, ‘Ice Cream and Frozen Desserts’ and ‘Soup’. Among the non-alcoholic beverages, all product categories were 100 % ultra-processed apart from ‘Juice’. Consequently, there was not enough variability in the model and no multiple linear regression was calculated for non-alcoholic beverages. The final multiple regression model for packaged foods included the CR4, a country fixed effect and a category fixed effect as predictor variables (Table 2). The product category ‘Rice, Pasta and Noodles’ was used as reference category as, on average, this was the least processed product category.

**Table 2:** Results of the two multiple linear regressions and the predictor variables included

Predictor variable	Regression	95 % CI
Intercept	17·03	9·21, 24·85
CR4	0·13	0·002, 0·25
Country fixed effect	Yes	
Product category fixed effect	Yes	

No significant correlations were detected between changes over the past 10 years in levels of market concentration and the proportion of sales of ultra-processed products (data not shown).

Simple linear regression analyses were performed to determine whether the number of companies per country with ≥1 % MS and the number of unique companies within packaged food and non-alcoholic beverage product markets significantly predicted the proportion of sales from ultra-processed products at country level in 2018.

Correlations of changes over time in the proportion of sales from ultra-processed products with changes in levels of market concentration were assessed. *R*-values >0·5 were considered to represent a strong correlation. *P*-values <0·05 were considered statistically significant.

All analyses were performed using Microsoft Excel and SAS 9.4 (2018).

## Results

The product categories ‘Asian Speciality Drinks’, ‘Carbonates’, ‘Concentrates’, ‘Confectionary’, ‘Energy Drinks’, ‘Ice Cream and Frozen Desserts’, ‘RTD Coffee’, ‘RTD Tea’, ‘Soup’ and ‘Sports Drinks’ were for 100 % ultra-processed across all European countries. Within the remaining twelve product categories, the proportion of ultra-processed sales varied per country. The level of market concentration, as measured by the CR4, varied per product category and country (Table 1). Several companies were included in the CR4 in multiple countries and across multiple product categories. Detailed information on the companies included in the CR4 of more than one product category as well as the number of countries in which the company was within the CR4 of this product category can be found in Annex 3.

### Market concentration and sales of less healthy products

A multiple linear regression model including the CR4, a country fixed effect and a product category fixed effect (Table 2) was significant and explained 93 % of the variance in sales of ultra-processed packaged foods (*F*(37 219) = 78·13, *P* < 0·0001).

The CR4 (*P* = 0·046), the country (*P* = 0·004) and the product category (*P* < 0·0001) were all significant predictors of sales of ultra-processed packaged food products. It was estimated that the proportion of sales of ultra-processed packaged food products increased with 0·13 for a one unit increase of the CR4, in addition to the increase caused by product category or the decrease caused by country, relative to the product category ‘Rice, Pasta and Noodles’ and the United Kingdom as reference country (Table 2, Annex 2). The fixed effect estimates, together with the *P*-values and 95 % CI, per product category and per country can be found in Annex 2.

### Market diversity and sales of less healthy products

**Fig. 1:**
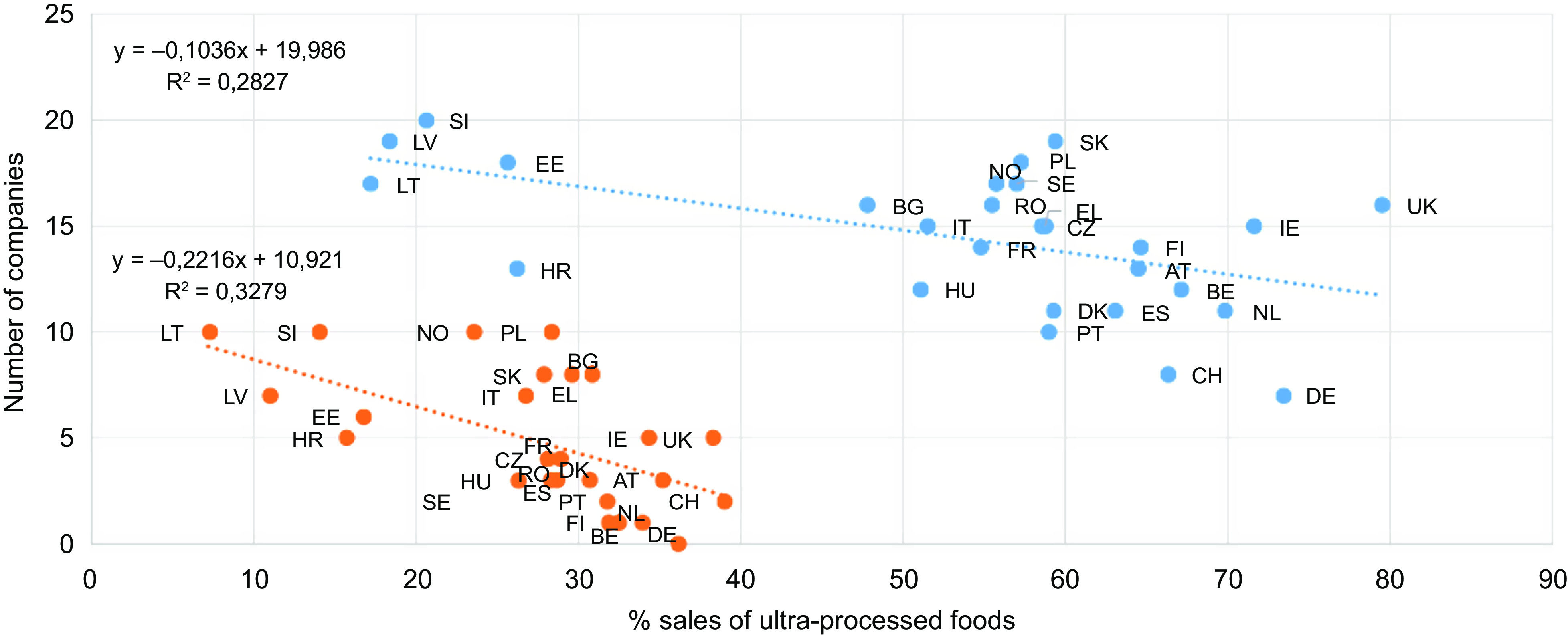
Regression of sales ultra-processed packaged food products (NOVA) with the number of companies with ≥1 % market share (MS) (blue) and the number of unique companies (orange). Countries are indicated to the right of the blue dots (the number of companies with ≥1 % MS) and to the left of the orange dots (the number of unique companies. NA means no sales of that category in that country in 2018 according to the Euromonitor data. Empty cells are countries for which no data were available at the most detailed level of the Euromonitor product categorisation system and as such products could not be classified. 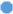, Companies with ≥ 1% market share; 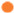, unique companies

The number of companies with ≥1 % MS and the number of unique companies per country both significantly predicted sales of ultra-processed packaged food products (*β* = -2·73, *P* = 0·004 and *β* = -3·06, *P* = 0·003, respectively). This was not the case for non-alcoholic beverages. Concretely, when per country the number of packaged food companies with ≥1 % MS and the number of unique packaged food companies increased, the sales of ultra-processed foods significantly decreased. Results are visually represented in Fig. 1.

## Discussion

This study set out to assess if market concentration, as measured by the CR4, and market diversity, assessed by the number of companies with ≥1 % MS and the number of unique companies per country, can predict the proportion of sales from ultra-processed products. A multiple linear regression model with the CR4, the country and the product category as predictor variables found that all three predictor variables significantly predicted the proportion of sales attributed to ultra-processed packaged food products. Increased market diversity in turn showed to significantly reduce sales of ultra-processed packaged food products but not non-alcoholic beverages. These results imply that increased market concentration, as measured by the CR4, may favour the increase in sales of ultra-processed packaged food products when taking into account both the product category and country. In contrast, increased market diversity in turn might be able to reduce sales of ultra-processed packaged food products.

Similar to our findings, a study in Asia found that market forces, including market concentration, were significant but variable drivers of the increase in sales of ultra-processed products. This study also observed that concentration was highest in ultra-processed product markets such as soft drinks, biscuits and snack foods^(22)^. This matches our finding that the product category had a strong effect in predicting sales of ultra-processed packaged food products.

A potential explanation for the decreased sales of ultra-processed products when more companies with ≥1 % MS and unique companies are present on the market could be that smaller companies lack both the financial and political resources to shape food environments and undermine public health^(3,23)^. Nonetheless, the sales of ultra-processed products is expanding worldwide, according to a study at global level using Euromonitor data^(4)^. To increase the healthiness of food environments, the food industry would need to reduce marketing and sales of ultra-processed products. This however inherently opposes the aim to maximise profits, especially for companies that rely on the sales of ultra-processed foods^(24,25)^. This conflict of interest may result in the food industry resorting to political activities to protect their markets and profitability^(4,9,24)^, something that becomes more attainable for dominant companies in highly concentrated markets with low market diversity^(9)^.

This study documents the possible impact of market structure on the healthiness of packaged foods and non-alcoholic beverages while highlighting the importance of looking beyond food policy to improve the healthiness of food environments. Nevertheless, this study has several limitations. Levels of market concentration may be an underestimation. The Euromonitor database focuses on brand ownership rather than companies. Consequently, companies that are considered independent in Euromonitor (and for the calculation of market concentration) may still sell brands from other companies through licensing agreements. Due to the lack of nutritional data at European level, there was insufficient variability to formulate conclusions for non-alcoholic beverages. Towards the future, more research is required using country-level data and detailed nutritional information to strengthen our understanding of the nutritional implications of market structures across Europe.

In conclusion, our results suggest that increased market concentration and reduced market diversity may predict increased sales of ultra-processed packaged food products across Europe. It is therefore recommended to take into account the market structure, in addition to policy effectiveness, when developing policies to improve food environments.

## Supplementary material

For supplementary material accompanying this paper visit https://doi.org/10.1017/S1368980022001926. click here to view supplementary material

## References

[1] Swinburn B , Sacks G , Vandevijvere S et al. (2013) INFORMAS (International network for food and obesity/non-communicable diseases research, monitoring and action support): overview and key principles: INFORMAS overview. Obes Rev 14, 1–12. 10.1111/obr.12087 24074206

[2] Swinburn B & Egger G (2004) The runaway weight gain train: too many accelerators, not enough brakes. BMJ 329, 736–739. 15388619 10.1136/bmj.329.7468.736 PMC518905

[3] Wood B , Williams O , Nagarajan V et al. (2021) Market strategies used by processed food manufacturers to increase and consolidate their power: a systematic review and document analysis. Glob Health 17, 17. 10.1186/s12992-021-00667-7 PMC7836045 33499883

[4] Baker P , Machado P , Santos T et al. (2020) Ultra-processed foods and the nutrition transition: global, regional and national trends, food systems transformations and political economy drivers. Obes Rev 21, e13126. 32761763 10.1111/obr.13126

[5] Monteiro CA , Moubarac JC , Levy RB et al. (2018) Household availability of ultra-processed foods and obesity in nineteen European countries. Public Health Nutr 21, 18–26. 28714422 10.1017/S1368980017001379 PMC10260838

[6] Monteiro CA , Cannon G , Levy R et al. (2016) NOVA. The star shines bright. World Nutr 7, 28–38.

[7] Hall KD , Ayuketah A , Brychta R et al. (2019) Ultra-processed diets cause excess calorie intake and weight gain: an inpatient randomized controlled trial of ad libitum food intake. Cell Metab 30, 77.e3. 10.1016/j.cmet.2019.05.020 PMC7959109 31269427

[8] Vandevijvere S , Jaacks LM , Monteiro CA et al. (2019) Global trends in ultraprocessed food and drink product sales and their association with adult body mass index trajectories. Obes Rev 20, 10–19. 31099480 10.1111/obr.12860

[9] Moodie R , Bennett E , Kwong EJL et al. (2021) Ultra-processed profits: the political economy of countering the global spread of ultra-processed foods – a synthesis review on the market and political practices of transnational food corporations and strategic public health responses. Int J Health Policy Manag 10, 968–982. 34124866 10.34172/ijhpm.2021.45 PMC9309965

[10] Popkin BM & Ng SW (2022) The nutrition transition to a stage of high obesity and noncommunicable disease prevalence dominated by ultra-processed foods is not inevitable. Obes Rev 23, e13366. 34632692 10.1111/obr.13366 PMC8639733

[11] Wood B , Williams O , Baker P et al. (2021) The influence of corporate market power on health: exploring the structure-conduct-performance model from a public health perspective. Glob Health 17, 41. 10.1186/s12992-021-00688-2 PMC8025506 33823900

[12] Pavic I , Galetic F & Piplica D (2016) Similarities and differences between the CR and HHI as an indicator of market concentration and market power. J Econ Manag Trade 13, 1–8.

[13] Chris P (2018) Market Concentration. Organisation for Economic Co-operation and Development. https://ssrn.com/abstract=3487657 (accessed June 2022).

[14] Van Dam I , Wood B , Sacks G et al. (2021) A detailed mapping of the food industry in the European single market: similarities and differences in market structure across countries and sectors. Int J Behav Nutr Phys Act 18, 54. 33902639 10.1186/s12966-021-01117-8 PMC8074488

[15] Gomes F & Lobstein T (2011) Food and beverage transnational corporations and nutrition policy. SCN News 39, 57–65.

[16] Clapp J & Scrinis G (2016) Big food, nutritionism, and corporate power. Globalizations 14, 578–595.

[17] Corfe S & Gicheva N (2017) Concentration Not Competition: The State of UK Consumer Markets. London: The Social Market Foundation.

[18] Falkner R (2003) Private environmental governance and international relations: exploring the links. Glob Environ Politics 3, 72–87.

[19] United Nations Children’s Fund (2019) *United Nations Special Rapporteur on the Right to Food. Protecting Children’s Right to a Healthy Food Environment*. Geneva: UNICEF and United Nations Human Rights Council. https://www.unicef.nl/files/Advocacy-brief-healthy-food-enviro-final.pdf (accessed June 2022).

[20] Euromonitor International (2017) Passport Global Market Information Database. http://www.portal.euromonitor.com (accessed June 2022).

[21] Naldi M & Flamini M (2014) The CR4 index and the interval estimation of the Herfindahl-Hirschman index: an empirical comparison. SSRN J, 1–11.

[22] Baker P & Friel S (2016) Food systems transformations, ultra-processed food markets and the nutrition transition in Asia. Glob Health 12, 1–15. 10.1186/s12992-016-0223-3 PMC5135831 27912772

[23] Baron DP (1995) Integrated strategy: market and nonmarket components. Calif Manag Rev 37, 47–65.

[24] Moodie R , Stuckler D , Monteiro C et al. (2013) Profits and pandemics: prevention of harmful effects of tobacco, alcohol, and ultra-processed food and drink industries. Lancet 381, 670–679. 23410611 10.1016/S0140-6736(12)62089-3

[25] Mialon M , Swinburn B & Sacks G (2015) A proposed approach to systematically identify and monitor the corporate political activity of the food industry with respect to public health using publicly available information. Obes Rev 16, 519–530. 25988272 10.1111/obr.12289

