# Supplementary material for: Market concentration and the healthiness of packaged food and non-alcoholic beverage sales across the European single market
Source: Public Health Nutr. 2022 Sep 8;25(11):3131–6. doi: 10.1017/S1368980022001926 (PMC9991654; doi:10.1017/S1368980022001926)
Supplement: Supplementary file 1 [file S1368980022001926sup001.docx]

**Annexes**

Annex 1: Euromonitor product subcategories per Euromonitor product category (indicated in orange) and their respective classifications according to the NOVA-classification. *RTE= Ready-To-Eat;* *RTD= Ready-To-Drink.*

| **Euromonitor food groups** | **NOVA** | **Euromonitor food groups** | **NOVA** |
| --- | --- | --- | --- |
| **Edible Oils** | | **Savoury Snacks** | |
| **Edible Oils** | Non-ultra-processed | **Nuts, Seeds and Trail Mixes** | Non-ultra-processed |
| **Ready meals** | | **Potato Chips** | Ultra-processed |
| **Shelf Stable Ready Meals** | Ultra-processed | **Tortilla Chips** | Ultra-processed |
| **Chilled Lunch Kits** | Ultra-processed | **Puffed Snacks** | Ultra-processed |
| **Chilled Pizza** | Ultra-processed | **Rice Snacks** | Ultra-processed |
| **Chilled Ready Meals** | Ultra-processed | **Vegetable, Pulse and Bread Chips** | Ultra-processed |
| **Dinner Mixes** | Ultra-processed | **Savoury Biscuits** | Ultra-processed |
| **Dried Ready Meals** | Ultra-processed | **Popcorn** | Ultra-processed |
| **Frozen Pizza** | Ultra-processed | **Pretzels** | Ultra-processed |
| **Frozen Ready Meals** | Ultra-processed | **Other Savoury Snacks** | Ultra-processed |
| **Prepared Salads** | Non-ultra-processed |  |  |
| **Sauces, dressings and condiments** | | **Sweet Biscuits, Snack Bars and Fruit Snacks** | |
| **Gravy Cubes and Powders** | Ultra-processed | **Dried Fruit** | Non-ultra-processed |
| **Liquid Stocks and Fonds** | Ultra-processed | **Processed Fruit Snacks** | Ultra-processed |
| **Stock Cubes and Powders** | Ultra-processed | **Cereal Bars** | Ultra-processed |
| **Dry Sauces** | Ultra-processed | **Energy Bars** | Ultra-processed |
| **Herbs and Spices** | Non-ultra-processed | **Fruit and Nut Bars** | Non-ultra-processed |
| **Monosodium Glutamate** | Ultra-processed | **Other Snack Bars** | Ultra-processed |
| **Pasta Sauces** | Ultra-processed | **Chocolate Coated Biscuits** | Ultra-processed |
| **Cooking Sauces** | Ultra-processed | **Cookies** | Ultra-processed |
| **Dips** | Ultra-processed | **Filled Biscuits** | Ultra-processed |
| **Pickled Products** | Ultra-processed | **Plain Biscuits** | Ultra-processed |
| **Barbecue Sauces** | Ultra-processed | **Wafers** | Ultra-processed |
| **Fish Sauces** | Ultra-processed | **Baked Goods** | |
| **Ketchup** | Ultra-processed | **Packaged Flat Bread** | Non-ultra-processed |
| **Mayonnaise** | Ultra-processed | **Unpackaged Flat Bread** | Non-ultra-processed |
| **Mustard** | Ultra-processed | **Packaged Leavened Bread** | Ultra-processed |
| **Oyster Sauces** | Ultra-processed | **Unpackaged Leavened Bread** | Non-ultra-processed |
| **Salad Dressings** | Ultra-processed | **Packaged Cakes** | Ultra-processed |
| **Soy Sauces** | Ultra-processed | **Unpackaged Cakes** | Non-ultra-processed |
| **Chili Sauces** | Ultra-processed | **Dessert Mixes** | Ultra-processed |
| **Other Table Sauces** | Ultra-processed | **Frozen Baked Goods** | Ultra-processed |
| **Tomato Pastes and Purées** | Ultra-processed | **Packaged Pastries** | Ultra-processed |
| **Yeast-based Spreads** | Ultra-processed | **Unpackaged Pastries** | Non-ultra-processed |
| **Other Sauces, Dressings and Condiments** | Ultra-processed |  |  |
| **Soup** | | **Breakfast Cereals** | |
| **Shelf Stable Soup** | Ultra-processed | **Hot Cereals** | Non-ultra-processed |
| **Chilled Soup** | Ultra-processed | **Children's Breakfast Cereals** | Ultra-processed |
| **Dehydrated Soup** | Ultra-processed | **Flakes** | Ultra-processed |
| **Frozen Soup** | Ultra-processed | **Muesli and Granola** | Ultra-processed |
| **Instant Soup** | Ultra-processed | **Other RTE Cereals** | Ultra-processed |
| **Sweet Spreads** | | **Processed Fruit and Vegetables** | |
| **Honey** | Non-ultra-processed | **Shelf Stable Beans** | Non-ultra-processed |
| **Chocolate Spreads** | Ultra-processed | **Shelf Stable Fruit** | Non-ultra-processed |
| **Jams and Preserves** | Ultra-processed | **Shelf Stable Tomatoes** | Non-ultra-processed |
| **Nut and Seed Based Spreads** | Ultra-processed | **Shelf Stable Vegetables** | Non-ultra-processed |
| **Dairy** | | **Frozen Fruit** | Non-ultra-processed |
| **Butter** | Non-ultra-processed | **Frozen Processed Potatoes** | Ultra-processed |
| **Cooking Fats** | Non-ultra-processed | **Frozen Processed Vegetables** | Non-ultra-processed |
| **Margarine and Spreads** | Ultra-processed | **Processed Meat and Seafood** | |
| **Spreadable Processed Cheese** | Ultra-processed | **Shelf Stable Processed Red Meat** | Ultra-processed |
| **Other Processed Cheese** | Ultra-processed | **Shelf Stable Processed Poultry** | Ultra-processed |
| **Packaged Hard Cheese** | Non-ultra-processed | **Chilled Processed Red Meat** | Non-ultra-processed |
| **Unpackaged Hard Cheese** | Non-ultra-processed | **Chilled Processed Poultry** | Non-ultra-processed |
| **Soft Cheese** | Non-ultra-processed | **Frozen Processed Red Meat** | Non-ultra-processed |
| **Dairy Only Flavoured Milk Drinks** | Ultra-processed | **Frozen Processed Poultry** | Non-ultra-processed |
| **Flavoured Milk Drinks with Fruit Juice** | Ultra-processed | **Shelf Stable Seafood** | Ultra-processed |
| **Fresh Milk** | Non-ultra-processed | **Chilled Processed Seafood** | Non-ultra-processed |
| **Shelf Stable Milk** | Non-ultra-processed | **Frozen Processed Seafood** | Non-ultra-processed |
| **Goat Milk** | Non-ultra-processed | **Chilled Meat Substitutes** | Ultra-processed |
| **Powder Milk** | Non-ultra-processed | **Frozen Meat Substitutes** | Ultra-processed |
| **Soy Drinks** | Ultra-processed | **Shelf Stable Meat Substitutes** | Ultra-processed |
| **Other Milk Alternatives** | Ultra-processed | **Rice, Pasta and Noodles** | |
| **Sour Milk Products** | Non-ultra-processed | **Chilled Noodles** | Non-ultra-processed |
| **Drinking Yoghurt** | Ultra-processed | **Instant Noodle Cups** | Ultra-processed |
| **Flavoured Yoghurt** | Ultra-processed | **Instant Noodle Pouches** | Ultra-processed |
| **Plain Yoghurt** | Non-ultra-processed | **Plain Noodles** | Non-ultra-processed |
| **Chilled Dairy Desserts** | Ultra-processed | **Chilled Pasta** | Non-ultra-processed |
| **Shelf Stable Dairy Desserts** | Ultra-processed | **Dried Pasta** | Non-ultra-processed |
| **Chilled Snacks** | Ultra-processed | **Rice** | Non-ultra-processed |
| **Coffee Whiteners** | Ultra-processed | **Bottled Water** | |
| **Flavoured Condensed Milk** | Ultra-processed | **Carbonated Natural Mineral Bottled Water** | Non-ultra-processed |
| **Plain Condensed Milk** | Non-ultra-processed | **Carbonated Spring Bottled Water** | Non-ultra-processed |
| **Cream** | Non-ultra-processed | **Carbonated Purified Bottled Water** | Non-ultra-processed |
| **Flavoured Fromage Frais and Quark** | Non-ultra-processed | **Flavoured Bottled Water** | Ultra-processed |
| **Plain Fromage Frais and Quark** | Non-ultra-processed | **Functional Bottled Water** | Non-ultra-processed |
| **Savoury Fromage Frais and Quark** | Non-ultra-processed | **Still Natural Mineral Bottled Water** | Non-ultra-processed |
| **Confectionary** | | **Still Spring Bottled Water** | Non-ultra-processed |
| **Chocolate Pouches and Bags** | Ultra-processed | **Still Purified Bottled Water** | Non-ultra-processed |
| **Boxed Assortments** | Ultra-processed | **Carbonates** | |
| **Chocolate with Toys** | Ultra-processed | **Low Calorie Cola Carbonates** | Ultra-processed |
| **Countlines** | Ultra-processed | **Regular Cola Carbonates** | Ultra-processed |
| **Seasonal Chocolate** | Ultra-processed | **Lemonade/Lime** | Ultra-processed |
| **Tablets** | Ultra-processed | **Ginger Ale** | Ultra-processed |
| **Other Chocolate Confectionery** | Ultra-processed | **Tonic Water/Other Bitters** | Ultra-processed |
| **Bubble Gum** | Ultra-processed | **Orange Carbonates** | Ultra-processed |
| **Chewing Gum** | Ultra-processed | **Other Non-Cola Carbonates** | Ultra-processed |
| **Boiled Sweets** | Ultra-processed | **Concentrates** | |
| **Liquorice** | Ultra-processed | **Liquid Concentrates** | Ultra-processed |
| **Lollipops** | Ultra-processed | **Powder Concentrates** | Ultra-processed |
| **Medicated Confectionery** | Ultra-processed | **Juice** | |
| **Power Mints** | Ultra-processed | **Not from Concentrate 100% Juice** | Non-ultra-processed |
| **Standard Mints** | Ultra-processed | **Reconstituted 100% Juice** | Ultra-processed |
| **Pastilles, Gums, Jellies and Chews** | Ultra-processed | **Juice Drinks (up to 24% Juice)** | Ultra-processed |
| **Toffees, Caramels and Nougat** | Ultra-processed | **Nectars** | Ultra-processed |
| **Other Sugar Confectionery** | Ultra-processed | **Coconut and Other Plant Waters** | Non-ultra-processed |
| **Ice Cream and Frozen Desserts** | | **RTD Coffee** | |
| **Frozen Desserts** | Ultra-processed | **RTD Coffee** | Ultra-processed |
| **Frozen Yoghurt** | Ultra-processed | **RTD Tea** | |
| **Single Portion Dairy Ice Cream** | Ultra-processed | **Carbonated RTD Tea** | Ultra-processed |
| **Single Portion Water Ice Cream** | Ultra-processed | **Still RTD Tea** | Ultra-processed |
| **Unpackaged Ice Cream** | Ultra-processed | **Energy Drinks** | |
| **Bulk Dairy Ice Cream** | Ultra-processed | **Energy Drinks** | Ultra-processed |
| **Ice Cream Desserts** | Ultra-processed | **Sports Drinks** | |
| **Multi-Pack Dairy Ice Cream** | Ultra-processed | **Sports Drinks** | Ultra-processed |
| **Bulk Water Ice Cream** | Ultra-processed | **Asian Speciality Drinks** | |
| **Multi-Pack Water Ice Cream** | Ultra-processed | **Asian Speciality Drinks** | Ultra-processed |

Annex 2: Output multiple linear regression model to predict the proportion of sales of ultra-processed packaged food products, including the CR4 and a country fixed effect as well as a product category fixed effect.

Annex 3: Companies included in the calculation of the four firm concentration ratio (CR4) and active across more than one product category. The numbers in bold in the second column represent the number of product categories for which the food company is part of the CR4. The numbers in the remaining columns represent the number of countries in which the company is part of the CR4 for the specific product category. *Green indicates that the company was only in the CR4 of that product category in one EU country. Yellow indicates that the company was in the CR4 in more than one and less than 11 EU countries. Red indicates that it was in the CR4 for that product category in 11 or more EU countries.* *RTD= Ready-To-Drink.*

| **Number of countries in which the company is within the CR4 for the product category** | **Number of categories** | **Asian Speciality Drinks** | **Baked Goods** | **Breakfast Cereals** | **Carbonates** | **Concentrates** | **Confectionery** | **Dairy** | **Energy Drinks** | **Ice Cream and Frozen Desserts** | **Juice** | **Processed Fruit and Vegetables** | **Processed Meat and Seafood** | **RTD Coffee** | **RTD Tea** | **Ready Meals** | **Rice, Pasta and Noodles** | **Sauces, Dressings and Condiments** | **Savoury Snacks** | **Soup** | **Sports Drinks** | **Sweet Biscuits, Snack Bars and Fruit Snacks** | **Sweet Spreads** |
| --- | --- | --- | --- | --- | --- | --- | --- | --- | --- | --- | --- | --- | --- | --- | --- | --- | --- | --- | --- | --- | --- | --- | --- |
| *2 Sisters Food Group Ltd* | **2** | . | . | . | . | . | . | . | . | . | . | 1 | 3 | . | . | . | . | . | . | . | . | . | . |
| *Acqua Minerale San Benedetto SpA* | **2** | . | . | . | 1 | . | . | . | . | . | . | . | . | . | 3 | . | . | . | . | . | . | . | . |
| *Adam Foods SL* | **4** | . | 2 | . | . | . | . | . | . | . | . | . | . | . | . | . | . | . | . | 1 | . | 2 | 2 |
| *Adolf Darbo AG* | **2** | . | . | . | . | 1 | . | . | . | . | . | . | . | . | . | . | . | . | . | . | . | . | 1 |
| *Agra Industrier AS* | **4** | . | . | . | . | . | . | 1 | . | . | . | . | 1 | . | . | . | . | 1 | . | . | . | . | 1 |
| *Agrofert as* | **3** | . | 3 | . | . | . | . | 1 | . | . | . | . | 1 | . | . | . | . | . | . | . | . | . | . |
| *Agrokor dd* | **11** | . | 2 | . | 1 | . | . | 1 | . | 3 | 1 | 2 | 1 | . | 1 | 1 | . | 1 | . | . | 1 | . | . |
| *Agrolimen SA* | **3** | . | . | . | . | . | . | . | . | . | . | . | . | . | . | . | 2 | 1 | . | 4 | . | . | . |
| *Alka Co SRL* | **2** | . | . | . | . | . | . | . | . | . | . | . | . | . | . | . | . | . | 1 | . | . | 1 | . |
| *Andros SAS* | **2** | . | . | . | . | . | . | . | . | . | . | 1 | . | . | . | . | . | . | . | . | . | . | 3 |
| *Apetit Oyj* | **3** | . | . | . | . | . | . | . | . | . | . | 1 | 1 | . | . | . | . | . | . | 1 | . | . | . |
| *Ardo NV* | **2** | . | . | . | . | . | . | . | . | . | . | 3 | . | . | . | . | . | . | . | 1 | . | . | . |
| *Arimex UAB* | **2** | . | . | . | . | . | . | . | . | . | . | . | . | . | . | . | . | . | 1 | . | . | 1 | . |
| *Arla Foods Amba* | **3** | . | . | . | . | . | . | 5 | . | . | . | . | . | 1 | . | . | . | . | . | 2 | . | . | . |
| *Associated British Foods Plc* | **3** | . | 1 | 4 | . | . | . | . | . | . | . | . | . | . | . | . | . | . | . | . | . | . | 1 |
| *Atlantic Grupa dd* | **4** | . | . | . | 2 | 2 | . | . | . | . | . | . | . | . | . | . | . | . | 1 | . | 1 | . | . |
| *Atria Oyj* | **2** | . | . | . | . | . | . | . | . | . | . | . | 3 | . | . | 1 | . | . | . | . | . | . | . |
| *Balti Veski AS* | **2** | . | . | 1 | . | . | . | . | . | . | . | . | . | . | . | . | 1 | . | . | . | . | . | . |
| *Barba Stathis SA* | **2** | . | . | . | . | . | . | . | . | . | . | 1 | . | . | . | 1 | . | . | . | . | . | . | . |
| *Barilla Holding SpA* | **5** | . | 3 | . | . | . | . | . | . | . | . | . | . | . | . | . | 14 | 1 | 4 | . | . | 3 | . |
| *Bel, Groupe* | **2** | . | . | . | . | . | . | 2 | . | . | . | . | . | . | . | . | . | . | . | . | . | . | 2 |
| *Bell Holding AG* | **2** | . | . | . | . | . | . | . | . | . | . | . | 1 | . | . | 1 | . | . | . | . | . | . | . |
| *Bidcorp Group* | **3** | . | . | . | . | . | . | . | . | 2 | . | 1 | . | . | . | 1 | . | . | . | . | . | . | . |
| *Bofrost Dienstleistungs GmbH & Co KG* | **3** | . | . | . | . | . | . | . | . | 2 | . | 1 | . | . | . | . | . | . | . | 1 | . | . | . |
| *Bolletje BV* | **3** | . | 1 | . | . | . | . | . | . | . | . | . | . | . | . | . | . | . | 1 | . | . | 1 | . |
| *Bolton Group, The* | **2** | . | . | . | . | . | . | . | . | . | . | . | 2 | . | . | 1 | . | . | . | . | . | . | . |
| *Bonafarm Group* | **5** | . | . | . | . | . | . | 1 | . | . | . | . | 1 | 1 | . | 1 | . | . | . | 1 | . | . | . |
| *Bonduelle Groupe SA* | **2** | . | . | . | . | . | . | . | . | . | . | 18 | . | . | . | 1 | . | . | . | . | . | . | . |
| *Bounty Brands Holdings Ltd* | **2** | . | . | . | . | . | . | . | . | . | . | . | . | . | . | . | 1 | . | . | 1 | . | . | . |
| *Britvic Plc* | **4** | . | . | . | 1 | 5 | . | . | . | . | 2 | . | . | . | . | . | . | . | . | . | 1 | . | . |
| *Carlsberg A/S* | **4** | . | . | . | 2 | . | . | . | 5 | . | . | . | . | . | 2 | . | . | . | . | . | 2 | . | . |
| *Cereal Partners Worldwide SA* | **2** | . | . | 23 | . | . | . | . | . | . | . | . | . | . | . | . | . | . | . | . | . | 1 | . |
| *Cerealis SGPS SA* | **2** | . | . | 1 | . | . | . | . | . | . | . | . | . | . | . | . | 1 | . | . | . | . | . | . |
| *Chipita SA* | **3** | . | 8 | . | . | . | . | . | . | . | . | . | . | . | . | . | . | . | 2 | . | . | . | 3 |
| *Coca-Cola Co, The* | **5** | . | . | . | 27 | 2 | . | . | . | . | 13 | . | . | . | 17 | . | . | . | . | . | 24 | . | . |
| *Colian Holding SA* | **2** | . | . | . | . | . | . | . | . | . | . | . | . | . | . | . | . | . | 1 | . | . | 1 | . |
| *Conserve Italia - Consorzio Cooperative Conserve Italia scarl* | **3** | . | . | . | . | . | . | . | . | . | 1 | 1 | . | . | . | . | . | 1 | . | . | . | . | . |
| *Contec Foods SRL* | **2** | . | . | . | . | . | . | . | . | . | . | 1 | . | . | . | . | . | 1 | . | . | . | . | . |
| *Continental Foods Europe BVBA* | **3** | . | . | . | . | . | . | . | . | . | . | . | . | . | . | 1 | . | 1 | . | 5 | . | . | . |
| *Co-Ro Food A/S* | **2** | . | . | . | . | 2 | . | . | . | . | 1 | . | . | . | . | . | . | . | . | . | . | . | . |
| *Cristim 2 Prodcom SRL* | **2** | . | . | . | . | . | . | . | . | . | . | . | 1 | . | . | 1 | . | . | . | . | . | . | . |
| *Dana doo* | **2** | . | . | . | . | 1 | . | . | . | . | 1 | . | . | . | . | . | . | . | . | . | . | . | . |
| *Danish Crown Amba* | **3** | . | . | . | . | . | . | . | . | . | . | . | 4 | . | . | 1 | . | . | . | 1 | . | . | . |
| *Danone, Groupe* | **3** | . | . | . | . | . | . | 15 | . | . | 1 | . | . | . | 3 | . | . | . | . | . | . | . | . |
| *Dimitar Madjarov EOOD* | **2** | . | . | . | . | . | . | 1 | . | . | . | . | 1 | . | . | . | . | . | . | . | . | . | . |
| *Dobrogea Grup SA* | **2** | . | 1 | . | . | . | . | . | . | . | . | . | . | . | . | . | . | . | . | . | . | 1 | . |
| *Dr Schär AG/SpA* | **2** | . | 2 | 1 | . | . | . | . | . | . | . | . | . | . | . | . | . | . | . | . | . | . | . |
| *Druskininku Rasa UAB* | **2** | . | . | . | 1 | 1 | . | . | . | . | . | . | . | . | . | . | . | . | . | . | . | . | . |
| *Ebro Foods SA* | **2** | . | . | . | . | . | . | . | . | . | . | . | . | . | . | . | 10 | 1 | . | . | . | . | . |
| *Eckes-Granini Group GmbH* | **3** | . | . | . | . | 5 | . | . | . | . | 11 | . | . | . | 1 | . | . | . | . | . | . | . | . |
| *Eesti Pagar AS* | **2** | . | 2 | . | . | . | . | . | . | 1 | . | . | . | . | . | . | . | . | . | . | . | . | . |
| *Efko Frischfrucht & Delikatessen GmbH* | **2** | . | . | . | . | . | . | . | . | . | . | 1 | . | . | . | . | . | 1 | . | . | . | . | . |
| *Elbisco Group* | **2** | . | 1 | . | . | . | . | . | . | . | . | . | . | . | . | . | . | . | . | . | . | 1 | . |
| *Emco spol sro* | **2** | . | . | 2 | . | . | . | . | . | . | . | . | . | . | . | . | . | . | . | . | . | 1 | . |
| *Emmi Group* | **3** | . | . | . | . | . | . | 1 | . | 1 | . | . | . | 10 | . | . | . | . | . | . | . | . | . |
| *ETA Kamnik doo* | **2** | . | . | . | . | . | . | . | . | . | . | 1 | . | . | . | 1 | . | . | . | . | . | . | . |
| *European Food SA* | **3** | . | . | 1 | 1 | . | . | . | . | . | . | . | . | . | . | . | . | . | . | . | . | 1 | . |
| *Ferbar-Fernando Barros Lda* | **2** | . | . | . | . | . | . | . | . | . | . | 1 | . | . | . | . | . | 1 | . | . | . | . | . |
| *Ferrero & related parties* | **5** | . | 1 | . | . | . | 21 | . | . | . | . | . | . | . | 1 | . | . | . | . | . | . | 2 | 20 |
| *Fleury Michon, Groupe* | **2** | . | . | . | . | . | . | . | . | . | . | . | 1 | . | . | 3 | . | . | . | . | . | . | . |
| *Food Union Group* | **3** | . | . | . | . | . | . | 1 | . | 4 | . | . | . | . | . | 1 | . | . | . | . | . | . | . |
| *FoodCare Sp zoo* | **3** | . | . | 1 | . | . | . | . | 2 | . | . | . | . | . | . | . | . | . | . | . | 2 | . | . |
| *Frosta AG* | **2** | . | . | . | . | . | . | . | . | . | . | 1 | . | . | . | 1 | . | . | . | . | . | . | . |
| *Galinta ir Partneriai UAB* | **2** | . | . | 1 | . | . | . | . | . | . | . | . | . | . | . | . | 1 | . | . | . | . | . | . |
| *Gavrilovic doo* | **2** | . | . | . | . | . | . | . | . | . | . | . | 1 | . | . | 1 | . | . | . | . | . | . | . |
| *General Mills Inc* | **3** | . | . | . | . | . | . | 1 | . | 9 | . | . | . | . | . | 1 | . | . | . | . | . | . | . |
| *Glanbia Plc* | **3** | . | . | . | . | . | . | 1 | . | . | . | . | . | . | . | . | . | . | . | 1 | 2 | . | . |
| *Groupe d'Aucy* | **3** | . | . | . | . | . | . | . | . | . | . | 2 | . | . | . | 1 | . | . | . | 1 | . | . | . |
| *Hain Celestial Group Inc, The* | **3** | . | . | . | . | . | . | . | . | . | . | . | . | . | . | . | 1 | . | . | 2 | . | . | 1 |
| *Harboes Bryggeri A/S* | **3** | . | . | . | 1 | 1 | . | . | 1 | . | . | . | . | . | . | . | . | . | . | . | . | . | . |
| *Heineken NV* | **6** | . | . | . | 1 | 1 | . | . | 1 | . | 1 | . | . | . | 1 | . | . | . | . | . | 1 | . | . |
| *Hero Group GmbH* | **5** | . | . | . | . | . | . | . | . | . | . | 1 | . | . | . | 1 | 1 | . | . | . | . | 1 | 5 |
| *HKScan Oyj* | **2** | . | . | . | . | . | . | . | . | . | . | . | 4 | . | . | 1 | . | . | . | . | . | . | . |
| *Hortex Holding SA* | **3** | . | . | . | . | . | . | . | . | . | 1 | 3 | . | . | . | . | . | . | . | 1 | . | . | . |
| *Hug AG* | **2** | . | . | . | . | . | . | . | . | . | . | . | . | . | . | . | . | . | 1 | . | . | 1 | . |
| *Ion Cocoa & Chocolate Manufacturers SA* | **2** | . | . | . | . | . | 1 | . | . | . | . | . | . | . | . | . | . | . | . | . | . | . | 1 |
| *J García Carrión SA* | **2** | . | . | . | . | . | . | . | . | . | 1 | . | . | . | 1 | . | . | . | . | . | . | . | . |
| *Karl Fazer Oy Ab* | **3** | . | 5 | . | . | . | 3 | . | . | . | . | . | . | . | . | . | . | . | . | . | . | 2 | . |
| *Kavli Holding AS* | **4** | . | . | . | . | 1 | . | 1 | . | . | . | 1 | . | . | . | . | . | 1 | . | . | . | . | . |
| *Kellogg Co* | **3** | . | . | 22 | . | . | . | . | . | . | . | . | . | . | . | . | . | . | 9 | . | . | 1 | . |
| *Kendy Suisse AG* | **2** | . | . | . | . | 1 | . | . | . | . | . | . | . | . | . | . | . | . | . | 1 | . | . | . |
| *Kerry Group Plc* | **2** | . | . | . | . | . | . | 1 | . | . | . | . | 2 | . | . | . | . | . | . | . | . | . | . |
| *Klaipedos Duona UAB* | **2** | . | 1 | . | . | . | . | . | . | . | . | . | . | . | . | . | . | . | . | . | . | 1 | . |
| *Kofola SA* | **4** | . | . | . | 4 | 3 | . | . | 2 | . | . | . | . | . | 1 | . | . | . | . | . | . | . | . |
| *Kraa dd* | **2** | . | . | . | . | . | 1 | . | . | . | . | . | . | . | . | . | . | . | . | . | . | 2 | . |
| *Kraft Heinz Co* | **8** | . | . | 1 | . | 1 | . | . | . | . | 1 | 2 | . | . | . | 2 | 1 | 9 | . | 4 | . | . | . |
| *Kyknos Greek Canning Co SA* | **2** | . | . | . | . | . | . | . | . | . | . | 1 | . | . | . | . | . | 1 | . | . | . | . | . |
| *La Linea Verde Società Agricola SpA* | **2** | . | . | . | . | . | . | . | . | . | . | . | . | . | . | 1 | . | . | . | 1 | . | . | . |
| *Lactalis, Groupe* | **4** | . | . | . | . | . | . | 11 | . | 1 | 3 | . | . | 1 | . | . | . | . | . | . | . | . | . |
| *Lantmännen ek för* | **4** | . | 6 | 4 | . | . | . | . | . | . | . | . | . | . | . | . | 2 | . | 1 | . | . | . | . |
| *Lerum AS* | **2** | . | . | . | . | 1 | . | . | . | . | . | . | . | . | . | . | . | . | . | . | . | . | 1 |
| *Linea Nivnice as* | **2** | . | . | . | . | 1 | . | . | . | . | 1 | . | . | . | . | . | . | . | . | . | . | . | . |
| *Lotus Bakeries NV* | **2** | . | 1 | . | . | . | . | . | . | . | . | . | . | . | . | . | . | . | . | . | . | 2 | . |
| *MAAG Grupp AS* | **2** | . | . | . | . | . | . | 1 | . | . | . | . | 1 | . | . | . | . | . | . | . | . | . | . |
| *Mars Inc* | **5** | . | . | . | . | . | 21 | . | . | 7 | . | . | . | . | . | . | 13 | 4 | . | . | . | 1 | . |
| *Maspex Wadowice Grupa* | **9** | . | . | 1 | . | 3 | . | . | 1 | . | 8 | . | . | . | 1 | 1 | 1 | 1 | . | . | . | . | 1 |
| *McCain Foods Ltd* | **3** | . | . | . | . | . | . | . | . | . | . | 8 | 1 | . | . | 1 | . | . | . | . | . | . | . |
| *Mlinotest dd* | **2** | . | 1 | . | . | . | . | . | . | . | . | . | . | . | . | . | 1 | . | . | . | . | . | . |
| *Mondelez International Inc* | **9** | . | 2 | 1 | . | 1 | 25 | 1 | . | 2 | . | . | . | . | . | . | . | . | 9 | . | . | 25 | 1 |
| *Nectar doo* | **3** | . | . | . | . | 1 | . | . | . | . | 1 | . | . | . | 1 | . | . | . | . | . | . | . | . |
| *Nestlé SA* | **11** | 1 | . | . | 1 | . | 10 | . | . | . | . | 1 | 3 | 12 | 17 | 7 | 1 | 8 | . | 19 | . | . | . |
| *Nichols Plc* | **2** | . | . | . | 1 | 1 | . | . | . | . | . | . | . | . | . | . | . | . | . | . | . | . | . |
| *Nomad Foods Ltd* | **5** | . | . | . | . | . | . | . | . | 1 | . | 12 | 5 | . | . | 7 | . | . | . | 3 | . | . | . |
| *Oetker-Gruppe* | **5** | . | 4 | 5 | . | 1 | . | . | . | 2 | . | . | . | . | . | 16 | . | . | . | . | . | . | . |
| *Olvi Oyj* | **5** | . | . | . | 3 | 1 | . | . | 3 | . | 1 | . | . | . | . | . | . | . | . | . | 2 | . | . |
| *Orkla Group* | **15** | . | 2 | 1 | . | 5 | 4 | . | . | 1 | 3 | 7 | 4 | . | . | 5 | 5 | 11 | 7 | 7 | . | 5 | 7 |
| *OSM Lowicz* | **2** | . | . | . | . | . | . | 1 | . | . | . | . | . | 1 | . | . | . | . | . | . | . | . | . |
| *Otsuka Holdings Co Ltd* | **3** | . | . | 1 | . | 5 | . | . | . | . | . | . | . | . | . | . | . | . | . | . | 16 | . | . |
| *Papadopoulos EJ SA* | **3** | . | 1 | . | . | . | . | . | . | . | . | . | . | . | . | . | . | . | 1 | . | . | 1 | . |
| *Paulig Ab* | **4** | . | 1 | . | . | . | . | . | . | . | . | . | . | 3 | . | 1 | . | 7 | . | . | . | . | . |
| *PepsiCo Inc* | **8** | . | . | 9 | 26 | 1 | . | . | 3 | . | 10 | . | . | . | . | . | . | . | 18 | 3 | 20 | . | . |
| *Pfanner Getränke GmbH, Hermann* | **2** | . | . | . | . | . | . | . | . | . | 2 | . | . | . | 6 | . | . | . | . | . | . | . | . |
| *Pieno Zvaigzdes AB* | **2** | . | . | . | . | . | . | 1 | . | 1 | . | . | . | . | . | . | . | . | . | . | . | . | . |
| *Pladis Ltd* | **2** | . | . | . | . | . | . | . | . | . | . | . | . | . | . | . | . | . | 1 | . | . | 5 | . |
| *Podravka dd* | **9** | . | 1 | 2 | . | . | 1 | . | . | . | . | 1 | . | . | . | 2 | 2 | 2 | . | 3 | . | . | 2 |
| *Põltsamaa Felix AS* | **2** | . | . | . | . | 1 | . | . | . | . | 1 | . | . | . | . | . | . | . | . | . | . | . | . |
| *Premia Foods AS* | **3** | . | . | . | . | . | . | 1 | . | . | . | 1 | . | . | . | 1 | . | . | . | . | . | . | . |
| *Premier Foods Plc* | **4** | . | 1 | . | . | . | . | . | . | . | . | . | . | . | . | . | 1 | 2 | . | 1 | . | . | . |
| *Puratos Group NV* | **2** | . | . | . | . | 1 | . | . | . | . | . | . | . | . | . | . | . | . | . | . | . | . | 1 |
| *Raisio Oyj* | **2** | . | . | 1 | . | . | . | . | . | . | . | . | . | . | . | . | 1 | . | . | . | . | . | . |
| *Rauch Fruchtsäfte GmbH & Co OG* | **5** | . | . | . | 1 | . | . | . | . | . | 5 | . | . | 8 | 6 | . | . | . | . | . | 3 | . | . |
| *Red Bull GmbH* | **2** | . | . | . | . | . | . | . | 27 | . | . | . | . | . | 2 | . | . | . | . | . | . | . | . |
| *Romaqua Holdings SA* | **2** | . | . | . | 1 | . | . | . | . | . | 1 | . | . | . | . | . | . | . | . | . | . | . | . |
| *Royal FrieslandCampina NV* | **2** | . | . | . | . | . | . | 6 | . | . | . | . | . | 1 | . | . | . | . | . | . | . | . | . |
| *Royal Unibrew A/S* | **5** | . | . | . | 3 | . | . | . | 2 | . | 2 | . | . | . | 1 | . | . | . | . | . | 2 | . | . |
| *Saarioinen Oy* | **4** | . | . | . | . | . | . | . | . | . | . | . | 1 | . | . | 2 | . | . | . | 2 | . | . | 1 |
| *Salumificio Flli Beretta SpA* | **2** | . | . | . | . | . | . | . | . | . | . | . | 1 | . | . | 1 | . | . | . | . | . | . | . |
| *Salvest AS* | **3** | . | . | . | . | . | . | . | . | . | . | . | . | . | . | 1 | . | . | . | 1 | . | . | 1 |
| *Scandia Food SRL* | **3** | . | . | . | . | . | . | . | . | . | . | . | 1 | . | . | 1 | . | . | . | 1 | . | . | . |
| *Scandza AS* | **3** | . | . | . | . | . | . | 1 | . | . | . | . | . | . | . | . | . | . | 1 | . | . | 2 | . |
| *SHS Group* | **2** | . | . | . | 1 | . | . | . | . | . | . | . | . | . | . | . | . | . | . | . | . | . | 1 |
| *Sigma Alimentos SA de CV* | **2** | . | . | . | . | . | . | . | . | . | . | . | 4 | . | . | 1 | . | . | . | . | . | . | . |
| *Spitz GesmbH, S* | **2** | . | . | . | . | 1 | . | . | 1 | . | . | . | . | . | . | . | . | . | . | . | . | . | . |
| *Standard Investment Management BV* | **4** | . | . | . | . | 1 | . | . | . | . | 2 | . | . | . | 1 | . | . | . | . | . | 1 | . | . |
| *Stanic Beverages doo* | **2** | . | . | . | . | 1 | . | . | . | . | 1 | . | . | . | . | . | . | . | . | . | . | . | . |
| *Sumol+Compal SA* | **4** | . | . | . | 1 | 1 | . | . | . | . | 1 | 1 | . | . | . | . | . | . | . | . | . | . | . |
| *Suntory Holdings Ltd* | **6** | . | . | . | 7 | 6 | . | . | 3 | . | 6 | . | . | . | 1 | . | . | . | . | . | 5 | . | . |
| *Tartu Mill AS* | **2** | . | . | 1 | . | . | . | . | . | . | . | . | . | . | . | . | 1 | . | . | . | . | . | . |
| *Ter Beke NV* | **2** | . | . | . | . | . | . | . | . | . | . | . | 1 | . | . | 1 | . | . | . | . | . | . | . |
| *Tine SA* | **8** | . | . | . | . | . | . | 1 | . | 1 | 1 | . | . | 1 | 1 | 1 | . | . | . | 1 | 1 | . | . |
| *Unicer - Bebidas de Portugal SA* | **2** | . | . | . | 1 | . | . | . | . | . | . | . | . | . | 1 | . | . | . | . | . | . | . | . |
| *Unilever Group* | **11** | . | . | . | . | 3 | . | 1 | . | 27 | . | 1 | 2 | . | 20 | 2 | 4 | 24 | . | 23 | . | . | 3 |
| *United Soft Drinks BV* | **2** | . | . | . | . | 1 | . | . | . | . | . | . | . | . | . | . | . | . | . | . | 2 | . | . |
| *Valeo Foods Ltd* | **8** | . | . | . | . | . | . | . | . | . | 1 | 1 | . | . | . | . | 1 | 1 | 1 | 1 | . | 1 | 1 |
| *Valio Oy* | **4** | . | . | . | . | . | . | 3 | . | . | 1 | . | . | 1 | . | . | . | . | . | 1 | . | . | . |
| *Viciunai Group* | **2** | . | . | . | . | . | . | . | . | . | . | . | 2 | . | . | 1 | . | . | . | . | . | . | . |
| *VIFON - Vietnam Food Industries JSC* | **2** | . | . | . | . | . | . | . | . | . | . | . | . | . | . | . | 2 | . | . | 1 | . | . | . |
| *Vikonda Koncernas* | **4** | . | . | . | . | . | . | . | . | 1 | . | . | . | . | . | . | . | 1 | . | 1 | . | . | 1 |
| *Vindija dd* | **5** | . | . | . | . | . | . | 1 | . | . | 1 | . | 1 | 1 | . | . | . | . | . | . | 1 | . | . |
| *Vitamin Well AB* | **2** | . | . | . | . | . | . | . | 1 | . | . | . | . | 1 | . | . | . | . | . | . | . | . | . |
| *Vivartia SA* | **4** | . | . | . | . | . | . | 2 | . | . | 1 | . | . | 1 | 1 | . | . | . | . | . | . | . | . |
| *Vivatis Holding AG* | **3** | . | . | . | . | . | . | . | . | . | . | . | . | 2 | . | 1 | . | . | . | 1 | . | . | . |
| *Zott GmbH & Co KG* | **2** | . | . | . | . | . | . | 1 | . | . | . | . | . | 1 | . | . | . | . | . | . | . | . | . |
| *Zuegg SpA* | **2** | . | . | . | . | . | . | . | . | . | 1 | . | . | . | . | . | . | . | . | . | . | . | 1 |
